# Chronic Renal Vein Thrombosis With Preserved Renal Function Due to Protein S Deficiency: A Diagnostic Challenge in a Resource-Limited Setting

**DOI:** 10.7759/cureus.96621

**Published:** 2025-11-11

**Authors:** Sreenath Sreedharan, Mohandas M.K., Vismaya K.B., Nikhil Raju

**Affiliations:** 1 Nephrology, Government Medical College, Thiruvananthapuram, IND; 2 Urology, Government Medical College, Thiruvananthapuram, IND

**Keywords:** acute pyelonephritis, chronic thrombosis, inherited thrombophilia, protein s deficiency, renal vein thrombosis

## Abstract

Renal vein thrombosis (RVT) refers to a clinical condition in which formation of a thrombus occurs in the renal veins or their branches. Though rare, incidence of RVT is most common amongst adults with nephrotic syndrome as well as the newborn infants with either a volume contraction or an inherited thrombophilia. In this case report, we present a case of left chronic RVT with symptoms of acute pyelonephritis. On evaluation for the etiology of RVT, the patient was found to have protein S deficiency and opted for medical therapy. The patient regularly followed up as an outpatient with improvement of symptoms on medical therapy. This case highlights the implications of RVT in adulthood, emphasizing the need for early diagnosis and intervention, since late identification could result in significant morbidity. However, in cases where genetic studies are declined or not possible due to resource constraints, patients can be adequately optimized medically to improve overall quality of life and decrease morbidity and mortality associated with this untreated anomaly.

## Introduction

Renal vein thrombosis (RVT) stands at the intersection of vascular pathology and renal physiology, including both historical significance and modern clinical relevance. Its capacity to remain clinically silent until precipitating serious complications such as pulmonary embolism (PE) or acute renal dysfunction underscores its insidious nature. The fact that RVT was recognized in early medical writings, dating back to the time of Hippocrates and the Knidian physicians of Asia Minor, reflects its longstanding impact on human health, predating modern diagnostic capabilities by centuries.

In the modern era, it was first described by Rayer, a French nephrologist. Rayer’s definition of RVT (“thrombus in the renal vein or one of its tributaries”) laid the foundation for subsequent understanding of the disease [1]. Over time, the pathophysiological understanding of RVT has evolved from these early observations to incorporate its genetic underpinnings, such as Factor V Leiden mutation in neonatal presentations, and its close association with proteinuric states and hypercoagulable conditions [2]. This duality of ancient recognition and contemporary scientific insight highlights RVT as a timeless clinical entity, demanding both vigilance in detection and precision in management.

Acute RVT classically presents with flank pain, microscopic or gross hematuria, a decline in renal function, and occasionally a painful palpable abdominal mass, whereas chronic RVT typically manifests insidiously and often remains asymptomatic. Literature indicates that there is no clear distinction between acute and chronic RVT, nor between unilateral and bilateral disease [3].

The risk factor assessment for RVT includes the well-established components of Virchow’s triad: endothelial injury, venous stasis, and hypercoagulable states [4,5]. Endothelial injury is commonly observed in settings such as trauma, vasculitis, prolonged use of central venous catheters, and renal transplantation. Venous stasis typically occurs in situations involving volume contraction, including dehydration, hemorrhage, or gastrointestinal fluid loss due to vomiting and diarrhea, particularly in infants and children [5].

We present the case of a 63-year-old woman with a history of poorly controlled diabetes mellitus and hypertension who initially presented with symptoms indicative of acute pyelonephritis. Contrast-enhanced CT (CECT) subsequently revealed the presence of chronic RVT. This case illustrates the importance of recognizing RVT as a potential diagnosis even in atypical clinical scenarios and underscores the value of tailored management strategies in resource-limited environments or when financial constraints restrict access to advanced care.

## Case presentation

A 63-year-old woman with a background of long-standing, poorly controlled diabetes mellitus and hypertension presented with an acute febrile illness characterized by high-grade fever, chills, dysuria, multiple episodes of non-bilious vomiting, and bilateral flank pain. She denied hematuria or abdominal distension.

On initial evaluation, she appeared acutely ill and toxic. Her vital signs were notable for tachycardia (heart rate (HR) 120 bpm), hypertension (blood pressure (BP) 160/90 mmHg), pyrexia (101°F), respiratory rate of 22 breaths per minute, and oxygen saturation of 95% on room air. Physical examination revealed right-sided renal angle tenderness, with no additional systemic abnormalities.

Her past history was significant for multiple episodes of urinary tract infections (UTIs) over the preceding year, though none had been microbiologically confirmed. Laboratory investigations revealed marked leukocytosis (total leukocyte count (TLC) 22,000/µL; N90 L10), anemia (hemoglobin (Hb) 9.2 g/dL), thrombocytosis (platelet count 6.09 × 10⁵/µL), and a raised erythrocyte sedimentation rate (ESR) (60 mm/hr). Renal function parameters were as follows: blood urea 23 mg/dL, serum creatinine 1.18 mg/dL. Serum electrolytes showed mild hyponatremia (Na⁺ 132 mEq/L) and normal potassium levels (K⁺ 4.3 mEq/L) (Table 1).

**Table 1:** Laboratory and ancillary investigations Hb: Hemoglobin; TLC: Total leukocyte count; PT: Prothrombin time; INR: International normalized ratio; aPTT: Activated partial thromboplastin time; Na^+^: Sodium; K^+^: Potassium; AST: Aspartate aminotransferase; ALT: Alanine aminotransferase; ALP: Alkaline phosphatase; ANA IF: Antinuclear antibody by immunofluorescence; C ANCA: Cytoplasmic anti-neutrophil cytoplasmic antibodies; P ANCA: Perinuclear anti-neutrophil cytoplasmic antibodies; APLA: Antiphospholipid antibody; ECG: Electrocardiogram; ECHO: Echocardiography; HBsAg: Hepatitis B surface antigen; Anti HCV: Antibody to hepatitis C virus; ELISA: Enzyme-linked immunosorbent assay

Parameter	Result	Units & Reference Range
Hb	9.2	g/dl (12–15)
TLC	22,000	cells/mm³ (4,000–11,000)
Platelet Count	6.09	lac/mm³ (1.5–4.5)
PT / INR	12 / 1.1	sec / ratio (11–14 sec/0.8 – 1.2)
aPTT	25	sec (25–35)
Blood Urea	23	mg/dl (15–40)
Serum Creatinine	1.18	mg/dl (0.6–1.2)
Serum Sodium (Na⁺)	132	mEq/L (135–145)
Serum Potassium (K⁺)	4.3	mEq/L (3.5–5.0)
Total Bilirubin/Direct Bilirubin	0.4/0.1	mg/dl (0.3–1.2/0.0 – 0.3)
AST/ALT	20/22	U/L (< 40/< 40)
Total Protein/Albumin	7.2/4.3	g/L (6.0–8.0/3.5–5.0)
ALP	135	U/L (44–147)
ANA IF	Negative	-
C ANCA	Negative	-
P ANCA	Negative	-
APLA Profile	Negative	-
ECG	Normal sinus rhythm	-
2D ECHO	Normal biventricular function; no clots or vegetations	-
HIV/HBsAg/Anti-HCV ELISA	Negative	-

Urinalysis demonstrated trace proteinuria, abundant pus cells, and 1-2 red blood cells (RBCs)/high power field (hpf), without casts or crystals. Urine culture grew Klebsiella species, sensitive to cotrimoxazole, cefoperazone-sulbactam, piperacillin-tazobactam, and meropenem.

**Figure 1:**
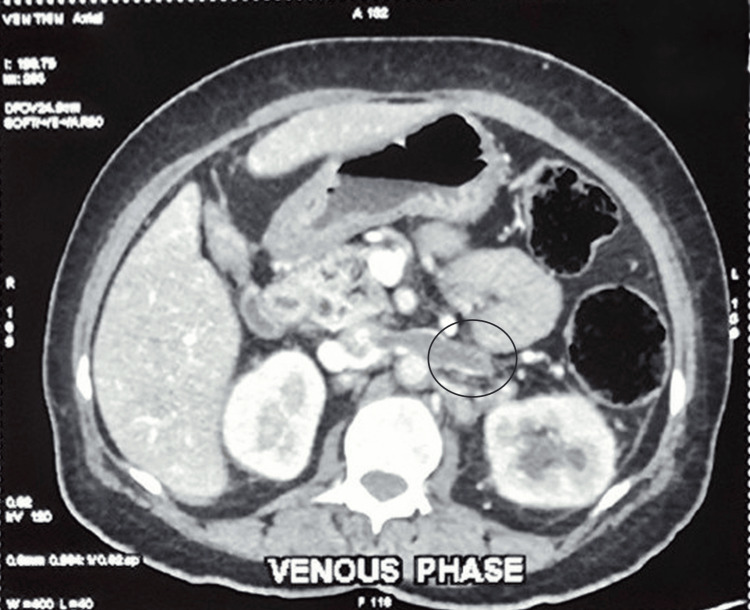
CECT venous phase showing left RVT CECT: Contrast-enhanced CT; RVT: Renal vein thrombosis

To rule out complicated UTI, a CECT of the abdomen and pelvis was performed, which showed focal edema in the form of swelling and lower attenuation of the lower pole of the left kidney, with perinephric stranding. A filling defect was noted in the left renal vein during the venous phase. There were no calculi or gas within the collecting system. The ureters and urinary bladder appeared normal. No malignant masses were identified in the abdomen or during screening of the thorax. The CECT abdomen was reported as radiologically consistent with features of bilateral pyelonephritis - left more than right - with left RVT (Figure 1). 

**Figure 2:**
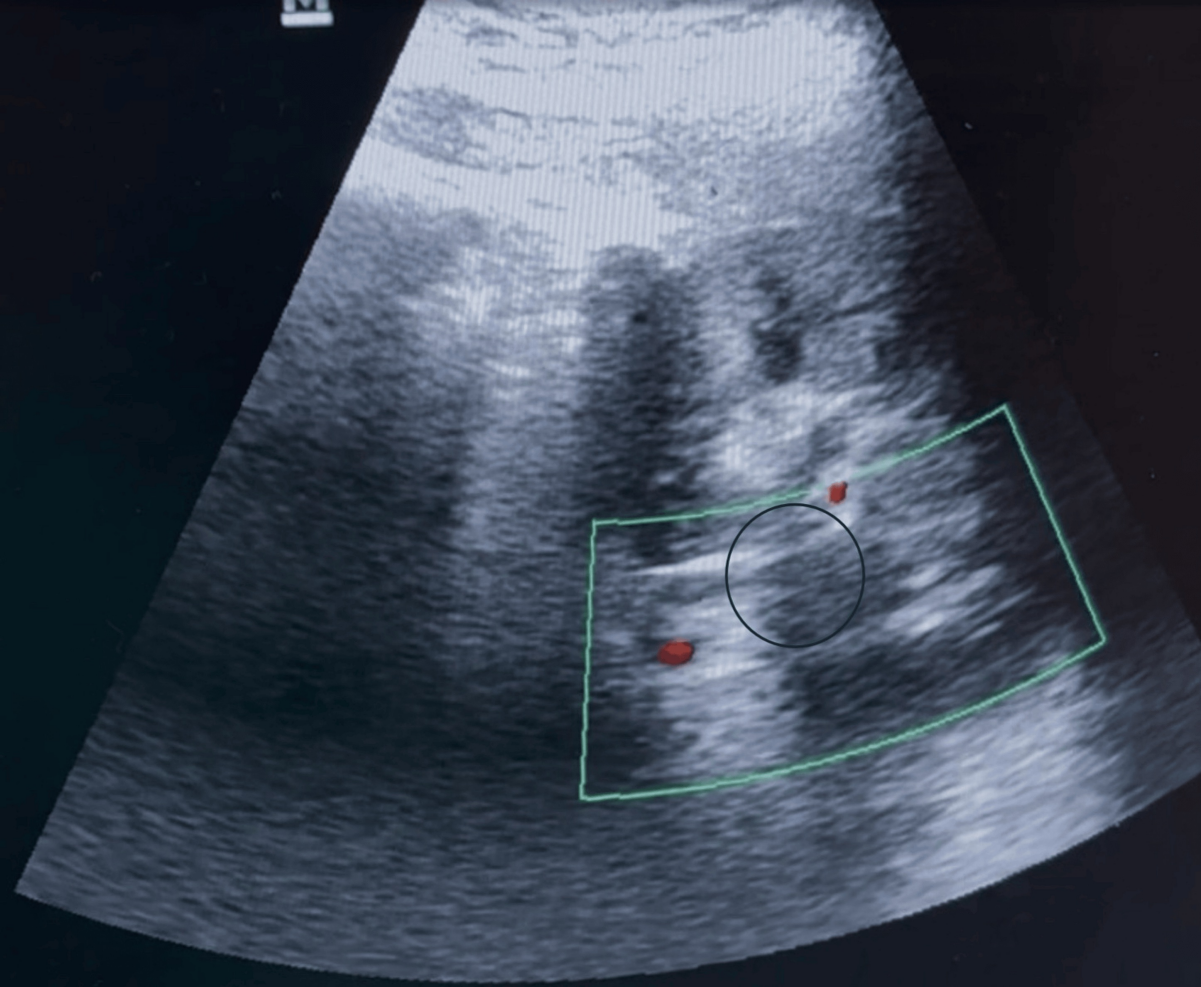
Renal Doppler image showing lumen occluding thrombus in the proximal left renal vein

Renal vein Doppler ultrasonography demonstrated a nearly circumferential eccentric echogenic thrombus causing luminal narrowing, with a thickness of 5.4 mm, in the left renal vein (Figure 2).

The patient was initiated on piperacillin-tazobactam injection and supportive medications for her complicated UTI, along with apixaban for RVT. An evaluation for the etiology of RVT ensued.

The patient had no history of blunt or surgical trauma, features of severe dehydration, or oral contraceptive pill use. There was no evidence of pedal oedema, hypoalbuminemia, or nephrotic-range proteinuria. She tested negative for HIV, hepatitis B virus (HBV), and hepatitis C virus (HCV). The coagulogram was within normal limits, and both antinuclear antibody (ANA) and antiphospholipid antibody (APLA) profiles were negative. A 2D echocardiogram revealed a normal study. Vasculitis workup, including perinuclear anti-neutrophil cytoplasmic antibodies (P ANCA) and cytoplasmic anti-neutrophil cytoplasmic antibodies (C ANCA), was also negative.

To explore rarer etiologies, we investigated levels of protein S, protein C, antithrombin, Factor V, and prothrombin gene mutations. All test results were normal except for protein S deficiency (24%, with a reference interval of 60-140%). Repeat testing confirmed these findings. Genomic studies could not be performed due to financial constraints and the unavailability of state-sponsored assays within the hospital.

After a literature review and discussion of possible treatment options with the patient, she was continued on tablet apixaban 5 mg twice daily. Expert opinion was sought from our interventional radiologists and vascular surgeons, who suggested reassessment with renal vein Doppler after six months.

Currently, the patient is on outpatient follow-up and remains asymptomatic.

## Discussion

Protein S deficiency is a hematologic disorder characterized by decreased levels or functional impairment of protein S: a vitamin K-dependent glycoprotein integral to the protein C anticoagulant pathway [5,6]. Beyond its well-established role in hemostasis, protein S exerts multifaceted effects on coagulation, inflammation, and apoptosis. Acting as an essential cofactor for activated protein C, it enhances the proteolytic inactivation of coagulation factors Va and VIIIa, thereby maintaining physiologic anticoagulant balance. Protein S circulates in two distinct forms: a free, biologically active fraction and a bound, inactive fraction complexed with C4b-binding protein. It is the free form that is primarily responsible for its anticoagulant activity.

Protein S deficiency can be either hereditary (autosomal dominant) or acquired (e.g., due to pregnancy, oral contraceptive use, or liver disease). Congenital protein S deficiency is primarily due to mutations in the PROS1 gene [7]. Hereditary protein S deficiency is classified into three types: Type I (quantitative deficiency), Type II (qualitative deficiency), and Type III (reduced free protein S levels and activity with normal total levels). Acquired fluctuations in protein S levels may arise from factors such as vitamin K antagonist (VKA) use, chronic infection, severe hepatic disease, systemic lupus erythematosus (SLE), myeloproliferative disorders, renal syndromes, disseminated intravascular coagulation, and oral contraceptive use [5].

Approximately half of patients with protein S deficiency present with symptoms before the age of 55 years [6]. Clinical manifestations of protein S deficiency include an increased risk of venous thromboembolism (VTE), purpura fulminans in neonates, and warfarin-induced skin necrosis [6,7]. VTE - including parenchymal thrombi, deep vein thrombosis (DVT), PE, and a predisposition to disseminated intravascular coagulation - is common, with some patients also developing cerebral, visceral, or axillary vein thrombosis. Rarely, RVT has also been associated with protein S deficiency [1,5]. Diagnosis of protein S deficiency is typically established through functional assays, such as clotting-based tests and enzyme-linked immunosorbent assays (ELISA), to assess protein S activity levels.

Management of VTE involves anticoagulation therapy with agents such as heparin (low-molecular-weight or unfractionated), VKAs, or direct oral anticoagulants (DOACs) [5]. The choice between a DOAC and a VKA is guided by patient preference and convenience. Although VKAs were historically the standard treatment for VTE, the advent of DOACs has shifted clinical practice toward their use. Owing to their favorable efficacy and safety profile, DOACs are now increasingly utilized in the management of VTE. In a cohort study involving patients with inherited thrombophilia, DOACs demonstrated comparable efficacy to heparin/VKA therapy. However, they were associated with a higher incidence of non-major bleeding, whereas VKAs showed a slightly greater risk of major bleeding [8]. In congenital protein S deficiency, anticoagulation therapy is typically continued for an extended duration, until coagulation parameters remain stable for at least two consecutive days [7].

This case emphasizes the importance of maintaining a high index of suspicion for RVT in older adults presenting with non-classical symptoms. There are limited reports of patients presenting with RVT secondary to protein S deficiency. Genetic studies to identify specific mutations could not be performed in our patient due to financial constraints. However, since the treatment consists primarily of anticoagulation for both inherited and acquired protein S deficiency, it was considered reasonable not to burden the patient with the additional cost of expensive gene sequencing analysis. We managed our patient with apixaban 5 mg twice daily and supportive care.

Our case reinforces the fact that even in the absence of genetic analysis, timely identification of RVT and appropriate etiological evaluation can lead to significant improvement in symptoms and overall quality of life.

## Conclusions

Protein S deficiency is an uncommon prothrombotic disorder characterized by reduced activity of protein S, a plasma serine protease with pivotal and multifaceted roles in coagulation, inflammation, and apoptosis. Although it most frequently manifests as VTE, notably DVT and PE, it can also give rise to thrombosis at atypical sites, including the renal and cerebral venous systems. The condition may be either hereditary or acquired.

Anticoagulation remains the cornerstone of management, with both VKAs and DOACs representing current therapeutic options. The choice between warfarin and a DOAC should be individualized, taking into account thrombotic burden, patient preference, adherence potential, and the risk of drug-drug or drug-diet interactions. Long-term surveillance with periodic clinical assessments and appropriate imaging is essential to monitor disease progression, treatment response, and recurrence risk.
